# Evidence needed for antimicrobial resistance surveillance systems

**DOI:** 10.2471/BLT.18.218917

**Published:** 2019-01-28

**Authors:** Cécile Aenishaenslin, Barbara Häsler, André Ravel, Jane Parmley, Katharina Stärk, David Buckeridge

**Affiliations:** a Research Group on Epidemiology of Zoonoses and Public Health, Faculty of Veterinary Medicine, Université de Montréal, 3200 Sicoitte, Saint-Hyacinthe, J2S 2M2, Quebec, Canada.; b Department of Pathobiology and Population Sciences, Royal Veterinary College, London, England.; c Public Health Agency of Canada, Guelph, Canada.; d SAFOSO AG, Liebefeld, Switzerland.; e Department of Epidemiology, Biostatistics and Occupational Health, McGill University, Montreal, Canada.

## Abstract

One Health surveillance for antimicrobial resistance has been promoted by the scientific community and by international organizations for more than a decade. In this article, we highlight issues that need to be addressed to improve the understanding of the effectiveness of One Health surveillance for antimicrobial resistance. We also outline the evidence needed to support countries planning to increase the level of integration of their surveillance system. Based on experience in Canada and other countries, we argue that more effort is needed to understand and measure the added value of One Health for antimicrobial resistance surveillance and to identify the most effective integration strategies. To date, guidelines for the development of One Health surveillance have focused mainly on the types of data that should be integrated. However, it may be necessary to apply the concept of One Health to surveillance tasks beyond data integration to realize the full value of the approach. Integration can be enhanced across different surveillance activities (data collection, analysis, interpretation and dissemination), taking account of the different skills and perspectives of experts and stakeholders involved. More research is needed to investigate the mechanisms through which a One Health approach to surveillance can increase the performance of antimicrobial resistance surveillance and, ultimately, improve health outcomes.

## Résumé

Cela fait plus de dix ans que la communauté scientifique et les organisations internationales préconisent l'application de l'approche «Un monde, une santé» à la surveillance de la résistance aux antimicrobiens. Cet article souligne les éléments à considérer pour mieux comprendre l'efficacité d'une surveillance fondée sur cette approche. Nous y évoquons également les données requises pour éclairer les pays dans la définition de leurs plans nationaux afin d'améliorer le niveau d'intégration de leur système de surveillance. À partir de l’expérience du Canada et d'autres pays, nous estimons que des efforts doivent encore être faits pour comprendre et mesurer la véritable valeur ajoutée de l'approche «Un monde, une santé» dans le cadre de la surveillance de la résistance aux antimicrobiens et afin d'identifier les stratégies d'intégration les plus efficaces. À ce jour, les lignes directrices pour l'établissement d'une surveillance fondée sur cette approche se sont principalement axées sur les types de données qui devraient être intégrées. Néanmoins, pour exploiter toute la valeur de cette approche, il pourrait être utile d'appliquer le concept «Un monde, une santé» aux activités de surveillance au-delà de la simple intégration des données. Une meilleure intégration peut être obtenue au niveau des différentes activités de surveillance (collecte, analyse, interprétation et diffusion des données) en tenant compte des différentes compétences et des différents points de vue des experts et des parties prenantes. De nouvelles recherches sont nécessaires pour comprendre les mécanismes par lesquels l'approche «Un monde, une santé» appliquée à la surveillance peut améliorer les performances de la surveillance de la résistance aux antimicrobiens et, en fin de compte, améliorer les résultats de santé.

## Resumen

La comunidad científica y las organizaciones internacionales han promovido durante más de una década la vigilancia sanitaria de la resistencia a los antimicrobianos. En este artículo, destacamos las cuestiones que deben abordarse para mejorar la comprensión de la eficacia de la vigilancia de la resistencia a los antimicrobianos de One Health. También esbozamos las pruebas necesarias para apoyar a los países que planean aumentar el nivel de integración de su sistema de vigilancia. Basándonos en la experiencia de Canadá y de otros países, sostenemos que se necesitan más esfuerzos para comprender y medir el valor agregado de One Health para la vigilancia de la resistencia a los antimicrobianos y para identificar las estrategias de integración más eficaces. Hasta la fecha, las directrices para el desarrollo de vigilancia de One Health se han centrado principalmente en los tipos de datos que deben integrarse. Sin embargo, puede ser necesario aplicar el concepto de One Health a tareas de vigilancia que van más allá de la integración de datos para aprovechar todo el valor del enfoque. La integración puede mejorarse en las diferentes actividades de vigilancia (recopilación, análisis, interpretación y difusión de datos), teniendo en cuenta las diferentes competencias y perspectivas de los expertos y las partes interesadas. Se necesita más investigación para estudiar los mecanismos mediante los cuales un enfoque de vigilancia de One Health puede aumentar el rendimiento de la vigilancia de la resistencia a los antimicrobianos y, en última instancia, mejorar los resultados sanitarios.

## ملخص

تم الترويج لمراقبة One Health لمكافحة مضادات الميكروبات بواسطة المجتمع العلمي ومنظمات دولية منذ ما يزيد عن عقد من الزمان. نسلط في هذا المقال الضوء على القضايا التي بحاجة للتناول لتحسين فهم فعالية مراقبة One Health لمكافحة مضادات الميكروبات. كما نوضح الأدلة اللازمة لدعم الدول التي تخطط لزيادة مستوى التكامل في نظام المراقبة الخاص بها. وبناءً على التجربة الكندية وتجارب بلدان أخرى، فإننا نزعم أننا بحاجة إلى بذل المزيد من الجهد لفهم وقياس القيمة المضافة لنظام One Health لمراقبة مكافحة مضادات الميكروبات، ولتحديد أكثر استراتيجيات التكامل من حيث الفعالية. وحتى الآن، ركزت إرشادات تطوير نظام One Health للمراقبة بشكل أساسي على أنواع البيانات التي ينبغي إحداث التكامل فيما بينها. ومع ذلك، قد يكون من الضروري تطبيق مفهوم One Health على مهام المراقبة التي تتجاوز تكامل البيانات، وذلك لإدراك القيمة الكاملة لهذا النهج. يمكن تعزيز التكامل عبر أنشطة المراقبة المختلفة (جمع البيانات وتحليلها وتفسيرها ونشرها)، مع الأخذ في الاعتبار المهارات المختلفة ووجهات نظر الخبراء وأصحاب المصلحة المعنيين. هناك حاجة إلى مزيد من الأبحاث للتحقيق في الآليات التي يمكن من خلالها لنهج One Health للمراقبة أن يزيد من أداء مراقبة مكافحة مضادات الميكروبات، مما يؤدي في النهاية إلى تحسين النتائج الصحية.

## 摘要

十多年来，科学界和国际组织一直致力于对抗菌素耐药性进行“唯一健康”监测。在本文中，我们强调了需要解决的问题，旨在提高“唯一健康”监测对抗菌素耐药性有效性的认识。我们还对所需的证据进行了概述，以支持那些计划提高其监测系统整合水平的国家。根据加拿大及他国经验，我们认为需要付出更多努力来理解和衡量“唯一健康”监测对抗菌素耐药性的附加价值，并确定最有效的整合策略。迄今为止，制定“唯一健康”监测的指南主要侧重于应整合的数据类型。然而，可能有必要将“唯一健康”概念应用于数据整合之外的监测任务，以实现该方法的全部价值。可以通过不同的监测活动（数据收集、分析、解释和传播）加强整合，同时考虑到所涉专家和利益相关者的不同技能和观点。需进一步研究以调查提高抗菌素耐药性监测表现的“唯一健康”方法，并最终改善健康结果。

## Резюме

Система надзора за резистентностью к антимикробным препаратам One Health находит поддержку научного сообщества и международных организаций уже в течение более десяти лет. В этой статье освещаются проблемы, которые следует решить для улучшения понимания эффективности надзора за резистентностью к антимикробным препаратам One Health. Авторы также описывают характер подтверждений, необходимых для поддержки стран, планирующих повысить уровень интеграции своих надзорных систем. На основании опыта Канады и других стран оспаривается утверждение о том, что нужно больше усилий для понимания и измерения ценности системы надзора за резистентностью к антимикробным препаратам One Health и для выявления наиболее эффективных стратегий интеграции. На сегодня рекомендации по разработке системы надзора One Health в основном сосредотачивались на типах данных, которые следует интегрировать. Однако, чтобы оценить всю ценность подхода, может понадобиться применение концепции One Health к надзорным задачам, не ограничивающимся интеграцией данных. Интеграция может быть усилена между разного рода надзорной деятельностью (сбор данных, анализ, интерпретация и распространение) с учетом различия в навыках и точках зрения специалистов и партнеров, участвующих в работе системы. Требуются дополнительные исследования для изучения механизмов, посредством которых подход One Health к вопросам надзора может улучшить качество работы систем надзора за антимикробной резистентностью и, в конце концов, улучшить результаты работы системы здравоохранения.

## Introduction

Antimicrobial resistance is a global threat to human and animal health and has major economic impacts.^1^^,^^2^ Antimicrobials are used in animal production to prevent or treat infectious diseases and to promote growth.^3^ The total quantity of antimicrobials used in animals is projected to increase by 67%, from 63 151 to 105 596 tonnes, between 2010 and 2030 due to increasing demand for animal food products globally and to more intensive animal farming practices in several middle-income countries.^4^ The extent to which the use of antimicrobials in animals contributes to the risk of developing antimicrobial resistance in humans at a global scale is unknown. A growing number of studies, however, provide support for causal relationships between antimicrobial use and resistance in animals and in humans at the local and regional scale.^5^ Consequently, surveillance systems that integrate information about resistance of microorganisms circulating in humans, animals and the environment are needed to enhance our understanding of the complex epidemiology of antimicrobial resistance and to inform targeted action. This integrated approach is known as One Health surveillance.^6^ One Health surveillance for antimicrobial resistance has been promoted by international organizations for more than a decade and constitutes a central recommendation of the World Health Organization (WHO) *Global action plan on antimicrobial resistance*.^7^

To assist countries in the development of surveillance systems, the WHO Advisory Group on Integrated Surveillance of Antimicrobial Resistance released its new guidelines on the application of a One Health approach to surveillance of antimicrobial resistance in foodborne bacteria in 2017.^8^ The report recommends detailed minimal requirements that each country should implement for an effective One Health approach.

In this article, we discuss three important issues that must be addressed to improve our understanding of the global effectiveness of One Health surveillance for antimicrobial resistance and to support countries in the development of such systems. First, we emphasize the need to better understand the mechanisms through which a One Health integrated approach enhances the effectiveness and value of surveillance systems. Second, we argue that more emphasis should be placed on transdisciplinary teams and networks brought together by the One Health approach. Third, we highlight that research is still needed to develop and evaluate conceptual and analytical tools for measuring the degree of integration and estimating the effect of this integration on health outcomes. Each topic is discussed in detail in the following sections. Our views are informed by ongoing work by ourselves and others that aims to evaluate the added value of One Health for the surveillance of antimicrobial resistance at the human–animal interface in Canada, Europe and globally.^6^^,^^9^^–^^11^ We use the Canadian Integrated Program on Antimicrobial Resistance Surveillance to illustrate many of our points.

## The value of integration

The Advisory Group on Integrated Surveillance of Antimicrobial Resistance guidelines describe in detail the diverse types of data that should be collected and analysed in a system for surveillance of antimicrobial resistance in foodborne bacteria.^8^ The guidelines recommend the collection of standardized and comparable data on antimicrobial resistance in bacteria from retail food products and humans and, if possible, data on antimicrobial use in humans and food-producing animals. The guidelines provide suggestions about which animal species and types of production should be targeted in relation to the food consumption patterns in the human population and the expected prevalence of antimicrobial resistance in animal populations. The surveillance system “may be modified from year to year to capture multiple commodities”. The guidelines also recommend which bacteria and antimicrobials to consider and stress the need to include samples from the environment. These recommendations are made with the implicit assumption that more data are inherently beneficial, without providing strong evidence about the added value of this approach or any recommendations about how One Health surveillance systems should be evaluated. Readers of the guidelines are encouraged to use existing integrated surveillance systems, such as those of Canada, Denmark, the Netherlands, Norway, the Republic of Korea, Sweden and the United States of America, as models for the development of new programmes in other countries.^8^

However, even though these model surveillance systems are all considered to be highly integrated, their levels of, and approaches to, One Health integration differ in many ways. For example, the Canadian Integrated Program for Antimicrobial Resistance Surveillance is operated entirely within a federal public health organization: the Public Health Agency of Canada. The programme collects annual resistance data on three microorganisms that are pathogens of humans (*Salmonella* spp*., Campylobacter* spp.) or are sentinel bacteria (*Escherichia coli*) from three main animal groups (poultry, pigs and cattle). Data are collected at different points along the food-chain, including the farm, the abattoir and retail food stores.^12^ In contrast, the Swedish surveillance system divides its operations between the animal and human health sectors. The Swedish National Veterinary Institute operates the Swedish Veterinary Antibiotic Resistance Monitoring programme and the Public Health Agency of Sweden manages the Swedish Antibiotic Utilization and Resistance in Human Medicine programme; the results are published in a joint report.^13^ The Swedish system integrates resistance data from several animal pathogens, including from companion animals, and varies the targeted animal species over time. The surveillance systems in Denmark, the Netherlands, Norway, the Republic of Korea and the United States all have different structures and processes, different target microorganisms and antimicrobials, and different points of collaboration across sectors.^14^^–^^17^ With such diversity, it may be difficult for countries with little or no experience with antimicrobial resistance surveillance to identify priorities and to construct an informed development plan to establish an integrated One Health surveillance system. Moreover, despite years of experience with One Health surveillance in several countries, evidence is still lacking that the performance and cost–effectiveness of highly integrated surveillance systems are better than less integrated systems. We therefore conclude that there is a need to move from generic guidelines for One Health integration to empirical evidence that can guide the design of surveillance systems. To produce such evidence, research is needed to assess the added value of existing One Health surveillance systems for antimicrobial resistance.

## Designing surveillance systems

In the Advisory Group on Integrated Surveillance of Antimicrobial Resistance report, integrated surveillance of antimicrobial resistance in foodborne bacteria is defined as “the collection, validation, analyses and reporting of relevant microbiological and epidemiological data on antimicrobial resistance in foodborne bacteria from humans, animals, and food, and on relevant antimicrobial use in humans and animals.”^8^ This definition tends to reduce the concept of One Health to integration of data sets from different sources: humans, animals and food. Apart from recommendations on the types of data that should be collected, the report presents some examples of integrated analysis and reporting formats. However, there is no clear guidance on other essential dimensions of One Health systems, such as intersectoral collaboration and information sharing.^11^

Collecting data from humans, animals and the environment is crucial to the concept of One Health and constitutes an important first step towards an integrated approach. However, the interdisciplinary nature of One Health surveillance generates outcomes that go beyond the value of information produced from integrated data collection. Also, to capture the added value of One Health surveillance, we need to revisit how we characterize and measure the integration of a surveillance system. In Table 1, we illustrate how One Health integration applies to all activities of a surveillance system from data collection to analyses, interpretation and dissemination. In each activity, integration can be achieved at different points and to differing extents in terms of the type of data integrated; the operations and processes used to integrate the data; and the collaboration of stakeholders that are implementing or benefiting from the activities. The level of integration can therefore be conceptualized, measured and increased for each surveillance activity.

**Table 1:** Types of integration in a surveillance system for antimicrobial resistance in foodborne bacteria by surveillance activities

Surveillance activity	Integration of information	Integration in operations and processes	Integration of multiple institutions, disciplines and perspectives
Data collection^a^	Integration of antimicrobial resistance data from: • human, animal and environmental sources; • different animal species; • different production type within a species (for example organic versus other productions) • different collection points (farm, abattoir, retail meat, water, soil); • different microorganism species; • different antimicrobials; and • active and passive surveillance activities	Standardization across human and animal sources of: • laboratory methods for samples; and • measurement units used to analyse and report antimicrobial resistance and antimicrobial use	Integration of: • data collection in one local or national organization (versus multiple organizations involved in animal health and human health); and • sample analysis in one laboratory (versus different laboratories for animal health, human health or others)
	Integration of data on antimicrobial use and other risk factors (e.g. farm management practices)		
Data analyses and interpretation	Comparisons of data on antimicrobial resistance and antimicrobial use from: • human, animal and environmental sources; • different animal species; • different animal commodities; • different collection points; • sick and healthy animals and humans; • different geographical locations; and • over time	Use of more complex integrated statistical analysis (versus simple comparisons of trends in data from different sources)	Integration of data analysis and interpretation: • in one institution (versus different institutions in animal and human health); • in one team of analysts (versus different individuals or teams); and • based on the perspectives of experts in different disciplines and the stakeholders involved in data interpretation
	Analysis of the links between antimicrobial use, other risk factors, and antimicrobial resistance	Analysis of relationships in antimicrobial resistance trends: • across data from different sources; and • in relation to risk factors	
Surveillance information dissemination	Integration of information from different sources in reporting activities (versus separated by sources)	Reporting: • using one main harmonized format for animal health and human health end-users (versus multiple formats); • at the same time to human and animal health stakeholders through at least one activity (one report or one meeting); and • adapted to stakeholders from human and animal health (other reporting activities)	Dissemination of information: • coordinated by one institution (versus different institutions in charge of dissemination); and • based on the perspectives of experts in different disciplines and the stakeholders involved in dissemination

^a^ Includes sample collection, sample analyses and data centralization.

At the level of data collection, integration can be increased by sampling from different animal species, production types, points along the food-chain, locations in the environment and species of microorganisms; and according to risk factors. For example, the Canadian Integrated Program on Antimicrobial Resistance Surveillance has increased its level of integration gradually over the years by adding collection points along the animal production system. The programme started by integrating data on antimicrobial resistance from animal samples collected at abattoirs with data on antimicrobial resistance from sick animals and humans. Over the next 15 years the programme added other type of data, including antimicrobial resistance of organisms in farm samples and in retail meat samples from different provinces, and the use of antimicrobials both in animal production and human health.

Integration can also be increased by harmonizing sample and data analyses methods across sectors to enhance the comparability of the information produced; or by centralizing surveillance operations related to data collection across one or more institutions. At the level of data analysis and interpretation, integration can be enhanced by moving from simple comparisons of trends between animal and human data sets towards multivariable analysis that, for example, controls for the effect of different risk factors. Moreover, One Health integration can be increased by including multiple disciplines in the analyst team. For example, the Canadian programme team is composed of epidemiologists and experts in avian, swine and bovine medicine as well as public health. This One Health team is key for analysis and interpretation of the surveillance information in context, and for the dissemination of surveillance information to a broad and multidisciplinary network of end-users. 

**Fig. 1:**
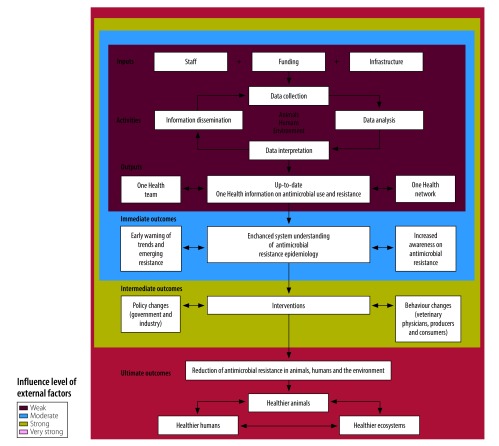
Logic model of a generic One Health surveillance system for antimicrobial resistance

Notes: The logic model illustrates the relationships between immediate, intermediate and ultimate expected outcomes of One Health surveillance. The arrows on the left present the growing strength of influence of external factors in achieving more distal outcome.

At the level of information dissemination, integration can be increased by moving from producing different reports for each animal species to integrating all surveillance information into a joint report, and by increasing the multidisciplinary nature of the network that is reached by these dissemination products.^18^ The Canadian programme team engages with over 600 stakeholders with expertise in animal and public health. Those involved include livestock and poultry producers; veterinary physicians; physicians and their licensing bodies; local, provincial and federal public health and animal health organizations; pharmaceutical organizations; drug and food regulators; animal and farm advocacy groups; and researchers. These stakeholders are invited to discuss integrated information on antimicrobial resistance at events, such as the annual stakeholder’s meeting of the Canadian Integrated Program on Antimicrobial Resistance Surveillance. The One Health network of stakeholders would not be able to exchange views and information to the same extent without the support of the Canadian programme team. Consequently, the surveillance team and its network of engaged stakeholders are essential to ensure that the information is used for decision-making and for inducing change in the use of antimicrobials in animal and human populations. Hence, we propose that the team and its network can be seen as direct contributors to the effectiveness of the One Health surveillance system (Fig. 1).

Integration in data analysis, interpretation and dissemination are important points in the design of effective One Health surveillance systems. We have developed a semiquantitative scale, based on our experience in the Canadian programme, to systematically measure the level of integration in antimicrobial resistance surveillance systems for foodborne bacteria. The scale comprises the elements that can be integrated in each surveillance activity, as we have already described, and proposes six levels of integration that can help to characterize One Health integration (Aenishaenslin C et al., Faculty of Veterinary Medicine, Université de Montréal, unpublished data, 2018). We hope that our scale will become an essential step in evaluating the effectiveness of One Health surveillance systems and serve as a guide for countries in the process of increasing the level of One Health integration within their surveillance system.

## Evaluating the added value

A greater level of integration in a system does not necessarily result in a more effective or more cost–effective system. Over recent decades, several generic frameworks for evaluation of surveillance systems in human and animal health have been developed by different researchers and organizations. The surveillance evaluation frameworks in both the public and animal health sectors propose groups of attributes that can be assessed as indicators of the effectiveness of a surveillance system, such as acceptability, data quality, data sensitivity, timeliness and cost–effectiveness.^19^^–^^21^ In line with these frameworks, researchers have used economic analysis to estimate the added value of One Health surveillance, although not to examine integrated surveillance of antimicrobial resistance.^22^^–^^25^

The value of One Health surveillance for antimicrobial resistance can be conceptualized and measured across a spectrum of different outcomes that can be classified as (i) immediate; (ii) intermediate; or (iii) ultimate (Fig. 1). Immediate outcomes include increased understanding of the antimicrobial resistance epidemiology at the interface across human health, animal health and the environment; the value of this knowledge is in the intellectual or social capital that is generated.^23^ Intermediate outcomes include changes in policy or behaviours resulting from surveillance information; the value in this case is in the size of the reduction in antimicrobial resistance that results from these changes. Only for the ultimate outcomes, improved animal, human and environmental health, can tangible benefits be measured directly in an economic evaluation. Hence, the benefits resulting from changes in the occurrence of disease in animals and humans can be attributed to decisions about interventions to mitigate the effect of disease (or decisions not to intervene) that were implemented because of surveillance outputs.^23^^,^^24^

Other researchers have conceptualized the objectives of One Health surveillance according to the epidemiological status of a disease within three stages of action to mitigate the disease: (i) sustainment; (ii) investigation; and (iii) implementation.^26^ The sustainment stage is when the disease incidence is at an acceptable level and mitigation efforts aim to keep the incidence under a certain threshold. In the investigation stage, the incidence goes over the acceptable level and the mitigation activities aim to collect the indicators (prevalence, morbidity, mortality, risk factors, etc.) that are necessary to identify the best points of intervention. Finally, in the implementation stage, the intervention is identified and implemented, while surveillance aims at evaluating its effectiveness.

Antimicrobial resistance is a complex and, for many countries, a relatively new concern. Surveillance information is therefore often not yet linked to planned interventions and the objectives of antimicrobial resistance surveillance mainly correspond to those in the investigation stage. The investigation stage can be maintained for years, as the complexity of the antimicrobial resistance epidemiology complicates identification of the most cost–effective interventions. Also, other types of changes that would lead to a reduction of antimicrobial resistance in animals, humans and their ecosystems, such as changes in policies and behaviours, are influenced by external factors, such as the political context (Fig. 1). Consequently, evaluation of the added value of One Health surveillance for antimicrobial resistance poses methodological challenges. An important step that has been neglected so far by researchers and public health practitioners is the evaluation of whether and how One Health approaches impact policy and behaviour changes. Mixed methods and qualitative methods, for example in policy analysis, should be considered to help understand how information produced by the surveillance system is used by different stakeholders and decision-makers.

## Conclusion

Countries developing One Health surveillance systems need empirical evidence about how to achieve integration in a cost–effective manner. Research on how the performance of One Health surveillance systems for antimicrobial resistance are influenced by different levels of integration is important for generating evidence and for benchmarking and recommending best practices.

Evidence about the added value of One Health surveillance systems is also important for effective implementation of the global antimicrobial resistance surveillance system.^27^ The system was launched in 2015 by WHO to standardize antimicrobial resistance surveillance across countries and help support the implementation of the global action plan on antimicrobial resistance.^28^ Up to now, the global system has focused on developing national surveillance systems able to produce information on antimicrobial resistance in humans that can be compared across countries. The global system also aims to support countries in moving towards surveillance systems capable of integrating data on antimicrobial resistance in animals. Countries will need evidence-based guidelines to structure the development of effective and efficient integrated surveillance for antimicrobial resistance. In addition, harmonized evaluation approaches and tools that are adapted to the complexity of One Health surveillance systems should be developed, integrated into the global antimicrobial resistance surveillance system and promoted at the international and national levels.

Evaluating the added value of One Health approaches for antimicrobial resistance surveillance is not a simple task, but it should not be set aside because of its complexity. We underline the need to better define and evaluate the components of One Health integration in antimicrobial resistance surveillance systems. Collaborations among One Health surveillance teams and their networks should be more carefully integrated in the design and evaluation of antimicrobial resistance surveillance systems and should be better addressed in guidelines provided by international organizations.
